# Recent advances in the use of TiO_2_ nanotube powder in biological, environmental, and energy applications

**DOI:** 10.1039/c9na00339h

**Published:** 2019-07-11

**Authors:** Walaa A. Abbas, Ibrahim H. Abdullah, Basant A. Ali, Nashaat Ahmed, Aya M. Mohamed, Marwan Y. Rezk, Noha Ismail, Mona A. Mohamed, Nageh K. Allam

**Affiliations:** Energy Materials Laboratory, School of Sciences and Engineering, The American University in Cairo New Cairo 11835 Egypt nageh.allam@aucegypt.edu

## Abstract

The use of titanium dioxide nanotubes in the powder form (TNTP) has been a hot topic for the past few decades in many applications. The high quality of the fabricated TNTP by various synthetic routes may meet the required threshold of performance in a plethora of fields such as drug delivery, sensors, supercapacitors, and photocatalytic applications. This review briefly discusses the synthesis techniques of TNTP, their use in various applications, and future perspectives to expand their use in more applications.

## Abstract 2

The use of titanium dioxide nanotubes in the powder form (TNTP) has been a hot topic for the past few decades in many applications.

## Graphical abstract

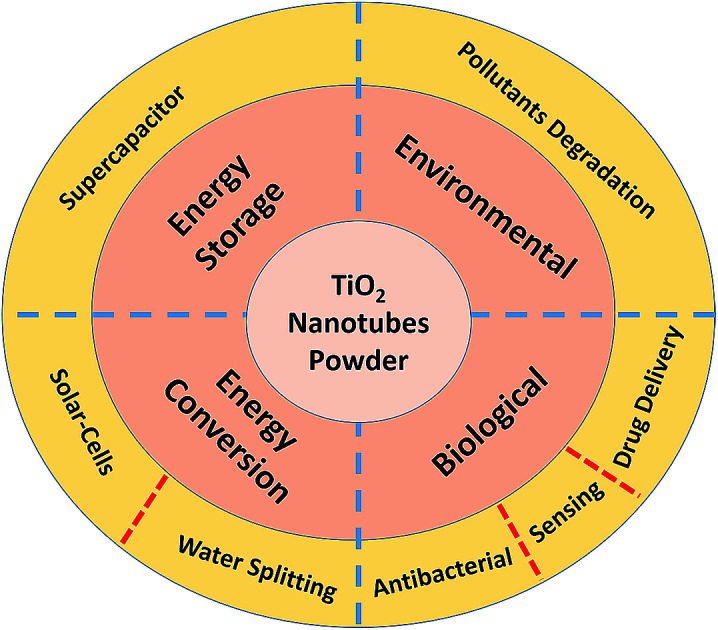

## Introduction

**Fig. 1:**
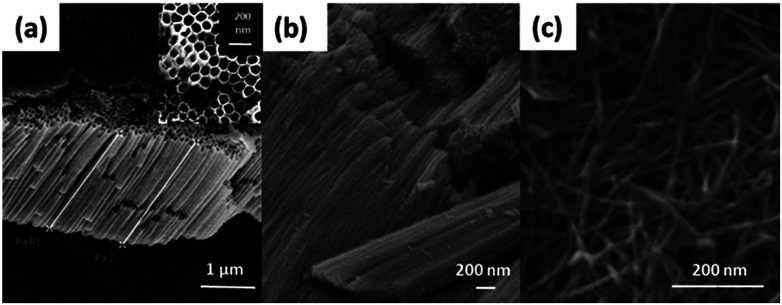
FESEM images of TNTP prepared by the authors *via* (a) ultra-sonication, (b) rapid breakdown anodization, and (c) hydrothermal techniques.

Nanomaterials with a tubular morphology enjoy unique properties over other morphologies, making them the target for many applications. Therefore, a plethora of fabrication techniques have been demonstrated in the literature to synthesize such nanotubes from different materials. Specifically, huge interest has been shown in the synthesis of titania nanotubes and their applications due to their biocompatibility,^1–3^ antimicrobial properties,^4,5^ high chemical stability, specific surface area, and catalytic activity.^6–8^ In addition, the high UV absorption and the possibility to modify the band gap promote titania as a good candidate for photocatalysis, making it useful for producing sunscreen materials^9^ and in water treatment.^10–13^ Of special interest, titania nanotubes in the powder form (TNTP) have recently gained great interest within the scientific community. To this end, many synthesis methods have been established to fabricate TNTP as shown in Fig. 1, including ultra-sonication after anodization, rapid breakdown anodization, and hydrothermal techniques. In this mini-review, the properties of TNTP will be highlighted by giving insights into their different synthesis techniques and use in a plethora of applications.

## Fabrication methods of TNTP

**Scheme 1:**
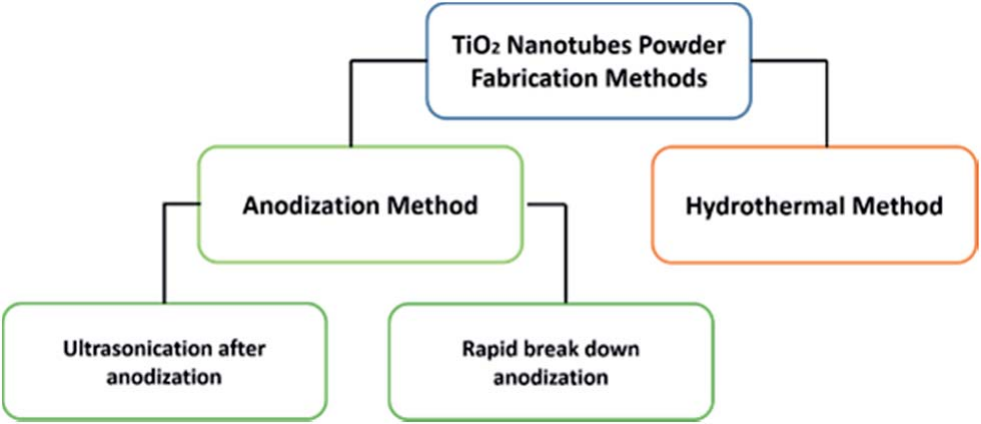
Fabrication methods of titania nanotubes in the powder form.

There are two main approaches to fabricate TiO_2_ nanotubes in the powder form as presented in Scheme 1. The first approach is the anodization of Ti foil, which can be subdivided into two techniques. While the fist approach includes the anodization of Ti foil followed by controlled ultrasonication,^14^ the second technique is a one-step process known as rapid breakdown anodization.^15,16^ The second approach is the hydrothermal synthesis of TNTP.^17,18^ Although there are other methods for producing tubular titania such as sol–gel and template-based synthesis methods,^19–21^ they are not commonly used.

## Anodization technique

**Fig. 2:**
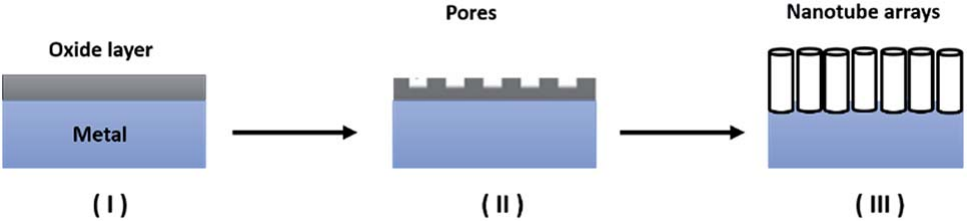
A schematic diagram of nanotube formation by anodization.

**Fig. 3:**
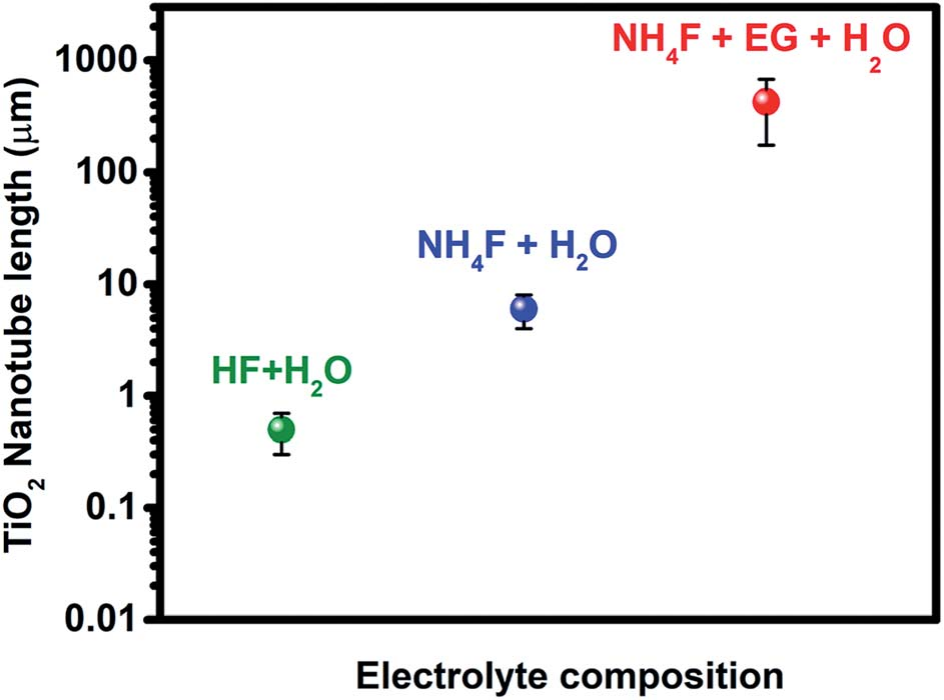
Effect of the electrolyte composition on the length of titania nanotubes formed during anodization.

The anodization process, a top-down fabrication technique, is an electrochemical method that produces an oxide layer on the surface of metals.^22^ In order to achieve the tubular array formation, there are three main processes: the first process is the field assisted oxidation of titanium metal to produce an oxide layer on its surface and to form TiO_2_. The second is the field assisted dissolution of titanium metal ions in the electrolyte. The final one is the surface etching resulting from the chemical dissolution of titanium and TiO_2_ as shown in Fig. 2.^3,19,23,24^ Extensive research studies have investigated the factors that govern the nanotube formation with tuned tube diameter and length.^25–29^ The formed oxide layer structure on the metal surface mainly depends on the concentration and composition of the electrolyte solution and the applied voltage.^5,8^ The effect of the electrolyte composition on the length of titania nanotubes is summarized in Fig. 3.

It has been reported that the formation of highly oriented TiO_2_ nanotubes with lengths ≅500 nm is achieved by the use of HF acidic aqueous electrolyte in the anodization of the titanium metal.^30^ Many researchers have paid attention to further synthesis approaches to enhance the titanium tube length and reduce the dissolution of the oxide layer on the surface of the Ti metal in a robust acidic medium. Therefore, several studies have been conducted to replace acidic HF electrolyte with fluoride salts such as NH_4_F, NaF, and KF at adjusted pH in order to increase the titanium nanotube length up to 6 μm.^31–37^ A novel approach was used to fabricate highly oriented titania nanotubes with a long tube length that reached up to 720 μm using the combination of non-aqueous organic electrolytes such as ethylene glycol (EG) or formamide (FA) with HF, KF, NaF, and NH_4_F.^19,30,32–37^

### Anodization and ultrasonication

Following the anodization of Ti foil, the TNTP can be formed *via* ultra-sonication of the pre-grown titania nanotubes followed by repetitive anodization and ultra-sonication processes until all the Ti foil has been fully converted into aligned nanotube powder. This process is also known as two-step anodization because the metal foil is used, recycled until it is fully consumed and converted into fine tubular powder.^14^

Although this method produces high surface area and well-defined structures of TiO_2_ tubular arrays, it has several drawbacks. In fact, it is considered time-consuming to extract the tubes and recycle the metal foil with an extremely small yield of the powder *via* ultra-sonication. In other words, the electrochemical reaction takes 2 hours per one cm^2^ of foil to produce only 0.01 g after annealing at 450 °C. In addition, due to the increase in temperature during ultra-sonication, the titania tubular architecture might be collapsed. Furthermore, the tubes are usually contaminated by electrolyte impurities, which could negatively affect their properties.^14^

### Rapid breakdown anodization

Using the rapid breakdown anodization technique to produce TNTP is considered the simplest and most cost-effective approach because it provides a high yield and can be achieved through a single electrochemical anodization step. The produced TNTP can be easily used in different applications due to their high surface area and aspect ratio. As discussed before, the formation mechanism of TNTP is mainly attributed to the chemical oxidation and dissolution of the metal substrate. In the rapid breakdown anodization, chloride ions are mainly used instead of fluoride ions in the electrolyte (*e.g.* perchloric acid).^14^ During the initial phase, the oxide layer is formed through the hydrolysis of the titanium metal surface. Once an electric field is applied, migration and transport of ions occur through the dissolution process, where Ti^4+^ cations migrate toward the electrolyte solution, and *via* the oxidation process, where oxygen anions diffuse towards the metal/oxide interface forming a thick oxide layer. After that, the electrolyte resistance is increased causing the anodic oxidation to stop. Then, the chloride anions start to dissolve the metal oxide layer forming pores which resulted from the localized breakdown of the oxide interface. The titanium dioxide white layer leaves the substrate and breaks down gradually in one dimension developing vertically oriented nanotubes in the electrolyte in the powder form as indicated by eqn (1)–(4).^15,16,38^

1 Ti + 2H_2_O → TiO_2_ + 4H^+^ + 4e^−^

2 2H_2_O → O_2_ + 4H^+^ + 4e^−^

3 Ti + O_2_ → TiO_2_

4 TiO_2_ + 4H^+^ + 6Cl^−^ → [TiCl_6_]^2−^ + 2H_2_O

**Fig. 4:**
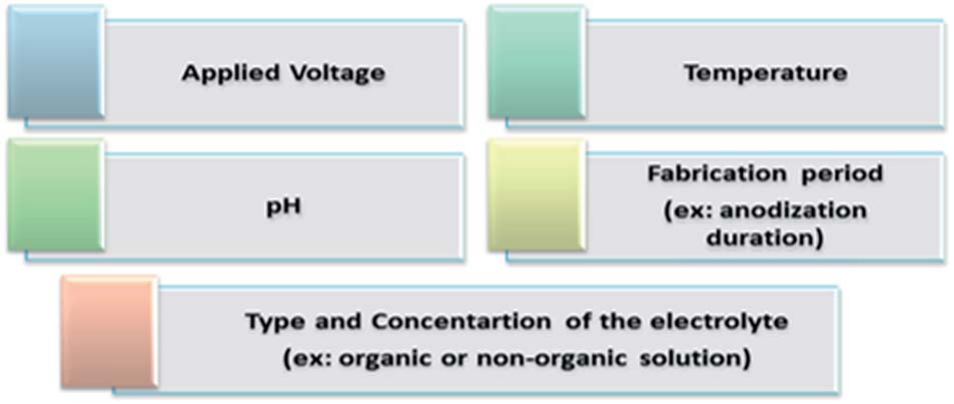
Factors affecting TNTP formation.

The chemical interactions explained above occur due to the mechanical stress established at the Ti/TiO_2_ interface. Moreover, the strong chemical reactions between the Ti substrate and chloride ions cause hydrogen evolution at the Pt electrode.^15,16^ A comparison between ultra-sonication, rapid breakdown anodization, and hydrothermal techniques is summarized in Table 1.^15,16^Fig. 4 summarizes the factors controlling the formation of TNTP such as the applied voltage, type and concentration of the electrolyte, temperature, pH, and fabrication processing period that definitely affects the tube diameter, tube length, etching rate, homogeneity, and roughness.^29^

**Table 1:** Comparison between the three main techniques for producing TiO_2_ nanotube powder (TNTP)

	Ultra-sonication technique	Rapid breakdown anodization	Hydrothermal technique
Advantages	Produces aligned nanotube arrays	Produces dispersed crystalline TiO_2_ NTs	Produces highly pure randomly aligned TiO_2_ NTs
			High surface area
	High surface area	High surface area	Cost-effective
	Cost-effective	Cost-effective	High yield
		High yield in a few minutes	Applicable on a large scale
Drawbacks	Under any circumstances, the tubular structure may collapse leading to reduced surface area	Need to rinse with DI water to ensure that TiO_2_ nanotubes are free of electrolyte impurities	Time-consuming (long processing time)
	Very low yield (0.01 g for 1 cm^2^ of foil)		Chemical-consuming
	Time-consuming		Produces non-uniform TNTs
	Repetitive and risky process, due to its two steps		Short length by default
	Electrolyte may contain impurities that adversely influence the biological applications		

### Hydrothermal processing

The hydrothermal process is used for crystal formation and growth.^39^ It is considered the most commonly used technique for the synthesis of TNTP due to its simplicity and high yield. Typically, amorphous TNTs are treated at high temperature in a concentrated sodium hydroxide solution.^40,41^ According to Moazeni *et al.*^42^ the formation of TNTP *via* hydrothermal processing involves six main steps. Initially, TiO_2_ and NaOH are mixed and stirred for 1 h and then subjected to ultra-sonication for another hour. The obtained suspension is then transferred to a Teflon-lined autoclave to be heated for 2 days. The resulting powder is washed and then aged in HCl to reach pH 2. The powder is then washed several times with deionized water and ethanol and dried at 40 °C for one whole day. It was noted that the alkaline solution caused some of the Ti and O bonds to be broken to form lamellar fragments as the growth mechanism was attributed to slow dissolution of TiO_2_ in a highly concentrated alkali solution. As titanate ions react with sodium from the alkali solution, they merge to form layered nanosheets. The induced mechanical stress caused by titanate ions at the borders of the sheets makes them scroll and wrap in the form of tubes.^42^ Zeng *et al.*^43^ used a similar technique to produce powder nanotubes. However, instead of treating TiO_2_ with NaOH at room temperature and stirring, they used NaOH solution inside a Teflon-lined autoclave at elevated temperature for 24 h. Upon subsequent cooling of the solution, it was titrated to reach the desired pH and dried. The obtained nanotubes have an outer diameter less than 10 nm and length less than 1 μm.^43^ Zavala *et al.*^39^ investigated the effect of hydrothermal treatment, annealing temperature, and acid washing on the morphology of TiO_2_ nanotubes. They realized that the hydrothermal treatment alters the TiO_2_ from the anatase to monoclinic phase. In addition, the temperature range between 400 °C and 600 °C maintained a highly stable tubular structure. Increasing the temperature above 600 °C resulted in the formation of irregular nanoparticles that are larger than the precursor TiO_2_ particle size. Moreover, the crystalline phase was changed from anatase to rutile. Finally, they proved the importance of acid washing as the exchange of Na^+^ ions promoted the formation of highly pure nanotubes.^18,39^ The hydrothermal processing is considered a relatively cost-effective method that produces highly pure TiO_2_ nanotubes. However, some drawbacks of the method should be taken into consideration, including non-uniformity, short length, and long synthesis time. However, it was shown that sonication pre-treatment would aid in increasing the length of the resulting nanotubes.^44^ Also, the stirring revolving speed was manipulated as a mechanical force to enhance the diffusion and the reaction rate of TiO_2_ nanocrystals to produce longer TNTP.^45,46^

## Applications of TNTP

**Fig. 5:**
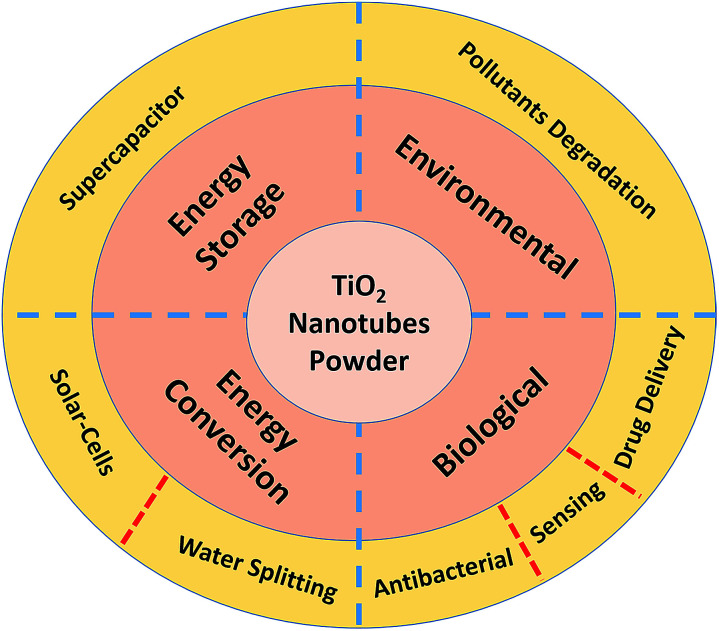
Selected TNTP applications.

Although TiO_2_ nanotubes in the powder form have been used in many applications, this review is focused on the specific applications shown in Fig. 5.

## Biological applications

### Drug delivery applications

**Fig. 6:**
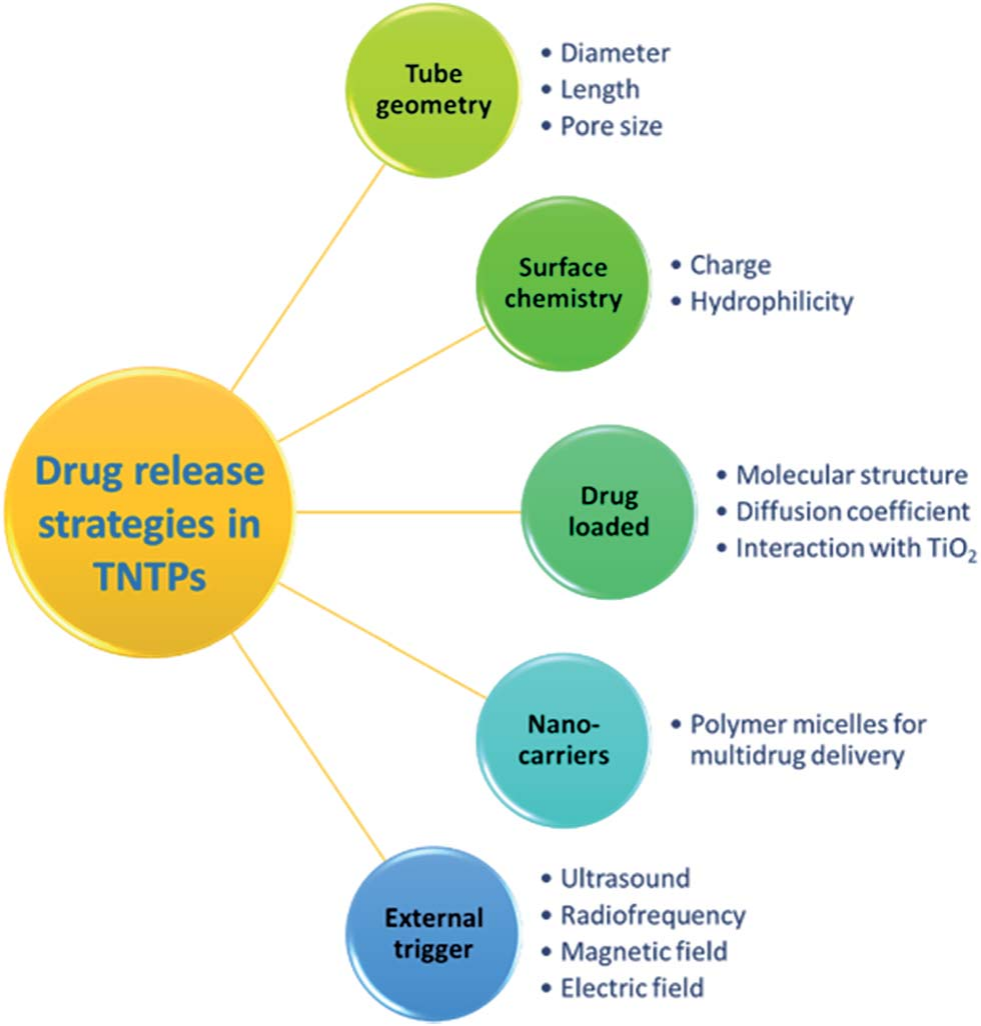
Governing strategies of drug release profiles using TNTP.

TiO_2_ nanotubes have been recently utilized to address the shortcomings of the conventional drug therapeutic solutions, particularly due to the excellent physicochemical properties and biocompatibility they possess.^3^ As current drug therapies may suffer from short circulating time, tedious pharmacodynamics, low resistance to the gastrointestinal system, and limited drug solubility, TNTP can help by providing an innovative delivery route for drugs to reach their target sites.^47^ It is worth noting that the diffusion process of TNTP when implanted in the body is governed by Fick's first law. This indicates that the drug release process will be dependent on several elements such as the nanotubes’ charge, dimensions, and surface chemistry, and the loaded drug’s charge, molecular size, and diffusion coefficient, as well as the type of interaction between the drug molecules and TiO_2_ inner surface, see Fig. 6.^48,49^ Accordingly, controlling the drug release profile is expected to depend on the fabrication and implementation conditions of TNTP. It is also of importance to mention that the most common drug release strategy is of the zero-order type, in which the release rate is constant regardless of the duration.^48^ In this regard, several studies tried to modify the nanotubular structure to suit the desired therapeutic strategy. These modifications include the adjustment of their length, thickness, pore opening, or stimulating their releasing process by polymeric coatings or other external sources.^50,51^ For instance, Aw *et al.* found that extending the tubular length from 25 to 100 μm resulted in an increase in the release duration for TiO_2_ nanotube drug delivery implants.^52^ Other types of drug release strategies consider varying dynamic change of the release kinetics, improving the drug loading and release patterns, multi-drug release, *etc.,* which were all pursued in numerous studies through functionalization of the nanotubular surface.^53,54^ For example, TiO_2_ nanotubes functionalized with 2-carboxyethyl-phosphonic acid and organic silanes such as penta-fluorophenyl dimethyl chlorosilane and 3-aminopropyl triethoxysilane have been utilized to modify the kinetics of both drug loading and release. This was obtained by changing the hydrophilic and hydrophobic properties of the nanotubular surface, which altered the interaction mechanism between the loaded drug and its carrier, the functionalized TiO_2_ nanotubes.^55^ For better controlled and sustained release profiles, several studies have reported exposing TiO_2_ nanotubes to external triggers such as ultrasound waves, radiofrequency, magnetic fields, and electric fields.^56^ As an example, the concept of ultrasound-sensitive systems of drug delivery has been proved by Aw *et al.* using TiO_2_ nanotubes. The drug-micelle release profile has shown a promising chance to be enhanced in accordance with the power intensity, pulse amplitude, length, and duration. This may be attributed to the combination of both cavitation and thermal processes triggered by ultrasonic waves. Accordingly, a better interaction between the loaded drug and TiO_2_ nanotubes is expected.^57^ This sort of modification would be of significant importance in local and complex delivery systems such as in brain and stent applications. Regarding the cytotoxicity effect of anodized TiO_2_ nanotubes on different types of cells, Li *et al.*^58^ have argued that the cytotoxicity of different nanostructures relies on their physiochemical factors such as size, shape, dose, surface charge, and chemical composition. In fact, the three main factors that influence the use of metal oxides in biomedical applications are size, shape, and dose.^58^ Chassot *et al.*^59^ performed a study to test the cytotoxicity of TiO_2_ nanotubes fabricated *via* the anodization method using protozoan *T. pyriformis* cells for *in vitro* studies. The research did not observe any cytotoxicity and confirmed that TiO_2_ nanotubes are not toxic.^59^ However, with all these possibilities and remarkable potential of TiO_2_ nanotube powder to be used in drug delivery systems, further *ex vivo* and *in vivo* animal studies are needed to examine the long-term tolerability and cytotoxicity of the material.

### Antibacterial applications

**Fig. 7:**
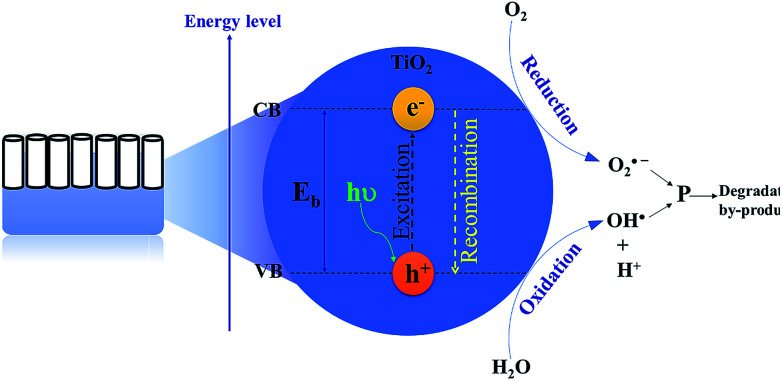
The principal mechanism for using TNTP in the process of water microbial disinfection.

Remarkable attention has been paid to the use of TiO_2_ nanomaterials in the field of photocatalytic bacterial disinfection.^60,61^ TNTP, as nanostructured semiconductor materials, are potent photoactive catalysts. They were utilized for eradicating harmful microorganisms and bacteria from water using solar irradiation.^62^ Using its different morphologies on the nano-scale, TiO_2_ has been proven to possess several advantages such as superior antimicrobial activity, high photo-stability (high corrosion resistance), biocompatibility, and strong photochemical oxidative activity. All these properties have qualified TiO_2_ as an excellent material for water microbial purification.^63^ Basically, the mechanism of water disinfection by TNTP relies on the hydroxylation reactions that start with the formation of hydroxyl radicals (OH˙). Upon light absorption by TNTP, the created electron–hole pairs trigger electrochemical redox reactions which produce free radicals such as hydroxyl radicals (OH˙).^64^ In the aqueous medium, these active radicals are strong enough to destroy the bacterial cell wall along with different other cellular components with extremely low survival levels as shown in Fig. 7.^65^ Typically, these OH˙ radicals are produced by the reactions of holes with either H_2_O molecules, their hydrolysed OH^−^ ions, or even bacterial membrane lipids.^66^ The radicals cause some deleterious effects on the extracellular medium of the bacteria, leading to serious chemical/biomolecular transformations. On the other hand, the electron counterparts combine with the proton ions (H^+^) in the same physiological environment to complete the other half of the electrochemical reaction.^67,68^

The effect of the concentration of titania in the lysogeny broth (LB) nutrient medium was tested against bacterial growth in drain water. The study by Carroll *et al.*^69^ has concluded that titania powder has the ability to diminish the growth of bacterial colonies even under dark conditions, with a reverse proportionality between the bacterial growth rate and the titania powder concentration.^69^ Interestingly, Abbas *et al.*^70^ have studied how different types of TNTP can deactivate the growth of *Escherichia coli* (*E. coli*) in contaminated water. The TNTP studied were prepared by both hydrothermal and rapid breakdown anodization techniques, along with other titania structures. The study has revealed that the hydrothermally synthesized TNTP were the best among other titania nanostructures, resulting in the highest inactivation rate of the *E. coli* bacteria under both dark and light conditions for 120 min.^70^ It was suggested that the hydrothermally prepared TNTP have a high abundance of –OH functional groups on their surfaces, mixed rutile and anatase phases, and remarkably high surface area. All these factors offered this particular structure the highest potential to result in the highest efficacy against bacterial growth in wastewater.^70^

## Energy conversion applications

### Solar cell applications

**Fig. 8:**
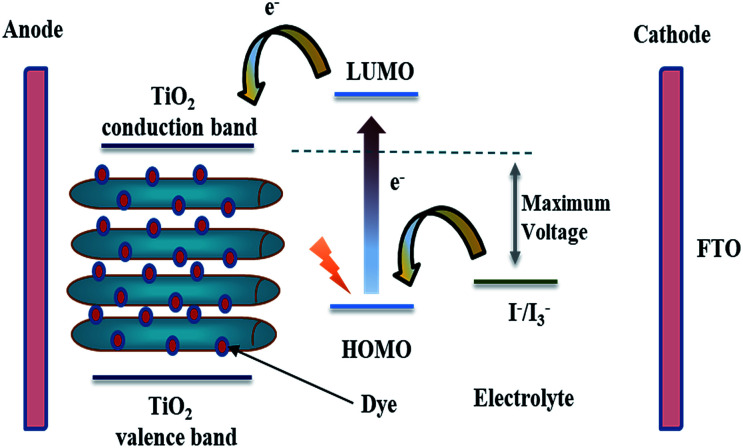
Schematic diagram of a DSSC using TNTP as an anode.

The vast majority of commercially available solar cells are made from silicon with different solid state junctions. The overall conversion efficiency is varied according to whether the silicon is mono- or multi-crystalline. Several approaches are being explored now in an attempt to achieve higher efficiencies with cost-effective materials. A promising photoelectrochemical concept is utilizing dye-sensitized TiO_2_ solar cells. In 1985, a Ru-based dye was adsorbed on TiO_2_ nanoparticles, which allowed the conversion of solar energy to electricity with 80% quantum efficiency.^71^ Later on, Grätzel implemented the concept to fabricate a full dye-sensitized solar cell (DSSC).^72^ The classic DSSC is mainly made of TiO_2_ crystalline nanoparticles attached to a conductive substrate, a Ru-based dye as a sensitizer, an electrolyte, and platinum as a counter electrode.^73^ A fundamental aspect of dye selection is that the LUMO of the dye has to be energetically higher than the TiO_2_ conduction band. Upon exposure to sunlight, the excited electrons of the dye are injected from the LUMO into the semiconductor's conduction band (see Fig. 8). The dye gets reduced through the redox reaction catalyzed by the electrolyte. The electrolyte is either an ionic liquid or an organic solvent. In addition to the crucial thermodynamic considerations, reaction kinetics have to be fulfilled, the electron injection from the semiconductor conduction band has to be faster than dye de-excitation, and also the dye regeneration time constant has to be fast enough to minimize any depletion effects.^74,75^ The struggle between effective electron transport within TiO_2_ and electron recombination possibilities is a limiting factor. Generally, TiO_2_ nanoparticles suffer from slow transport time constants owing to trapping/de-trapping effects. The hindered diffusion coefficient of the TiO_2_ nanoparticles is due to grain boundaries, defects, surface states, *etc.*, which drastically contribute to diminished electron flow as they act as trapping sites.^76,77^ In this regard, one-dimensional nanostructures such as TNTP can significantly improve the overall electron transport mechanism owing to limited inter-crystalline traps that lower the possibility of recombination. However, although many types of solar cells have been produced using 1D TiO_2_ morphologies, the multidirectional orientations of the misaligned 1D nanostructures do not guarantee the best unidirectional flow of electrons along the longitudinal length. The perfect 1D nanostructures provide rapid, conductive electron transport and the orientation becomes less important. This may apply for single crystalline nanostructures free from defects. Current approaches tend to grow polycrystalline TiO_2_ nanotubes, where a vertical alignment can compromise between electron transport and charge efficiency. Anodic oxidation approaches for synthesis of self-assembled titania nanotubes are becoming of great interest.^78,79^

The sole role of TiO_2_ in DSSCs is to harvest the injected electrons from the dye. Although other oxides could be considered as an alternative, until now TiO_2_ is the best choice. TNTs synthesized *via* rapid breakdown anodization (RBA) lead to the formation of flower-like bundles with very high aspect ratios when using perchlorate or chloride electrolytes. These TNTP show excellent performance in DSSCs.^80^ The TiO_2_ DSSC performance is mainly dictated by the degree of crystallinity. Upon elevating the temperature, the rutile phase dominates with the possibility of collapse. Thus, anatase is the phase of choice for efficient titanium-based solar cells as it is the most photoactive phase. Different groups have already reported several results but it is hard to compare these results because the overall produced efficiency is dictated by not only the intrinsic properties of the TiO_2_ NTs but also the entire solar cell structure *i.e.* the actually investigated active area within the solar cell, the distance between the nanotubes, and the counter electrode.^81^

The effects of combining TNTP and TiO_2_ nanoparticles prepared *via* sol–gel and hydrothermal methods, respectively, have been studied through measuring the performance of the solar cell. Various weight ratios of the TNTP and TiO_2_ nanoparticles were mixed together. The open TNTP structure facilitated better penetration of the electrolyte and enhanced the contact between the dye, tubes, and electrolyte. The high surface area of the nanotubes and the nanoparticles enhanced the amount of adsorbed dye. The crystal properties of the anatase phase were found to be the best at a hydrothermal temperature of 150 °C for 12 h. The overall conversion efficiency of the DSSC reached 4.56% under AM 1.5 illumination. It is worth mentioning that the photovoltaic performance of the DSSC made of hybrid titania nanoparticles and nanotubes is enhanced compared to that of the DSSC made purely of TiO_2_ nanoparticles.^82^ Also, the hybrid nanotubes and nanoparticles were tested in perovskite solar cells (PSCs). A (CH_3_NH_3_)PbI_3_ PSC based on TiO_2_ nanotube and nanoparticle hybrid photo-anode was successfully constructed without affecting the nanotubular structure. The charge efficiency was maximized and the recombination rates were suppressed. In this assembled device, the nanotubes boosted the light scattering and hence absorption by the sensitizer. The nanoparticles enhanced the adhesion of the cell components. Using carbon as a counter electrode, the conversion efficiency of the PSC reached 9.16% under 1.5 AM illumination.^83^ Hydrothermally annealed TNTP were sensitized with poly[2-methoxy-5-(2-ethylhexyloxy)-1,4-phenylenevinylene] (MEHPPV) as a conducting polymer used to improve the donor–acceptor mechanism of the hybrid solar cell. The different thermal treatments of the TiO_2_ nanotubes revealed drastic morphological, structural, electrical and optical alterations of the nanotubes, in addition to the remarkable induction of crystallinity and hence charge transfer enhancement. Here, the nanotubes act as an acceptor material and the MEHPPV polymer acts as a donor material, which improved the energy conversion of the organic solar cell.^84^ In an attempt to study the effect of different sensitizers on the efficiency of the TNTP, zinc porphyrin-imide dye was adsorbed on the TiO_2_ nanotubes by immersion for 24 h. The absorption spectra of the used zinc porphyrin-imide dye are usually seen at 439 nm and 620 nm. Upon adsorption on the TiO_2_ nanotubes, the peaks were shifted to 421 nm and 640 nm. The assembled DSSC showed a conversion efficiency of 1.914% from the front side and 1.147% from the backside.^85^

### Photocatalytic water splitting

**Fig. 9:**
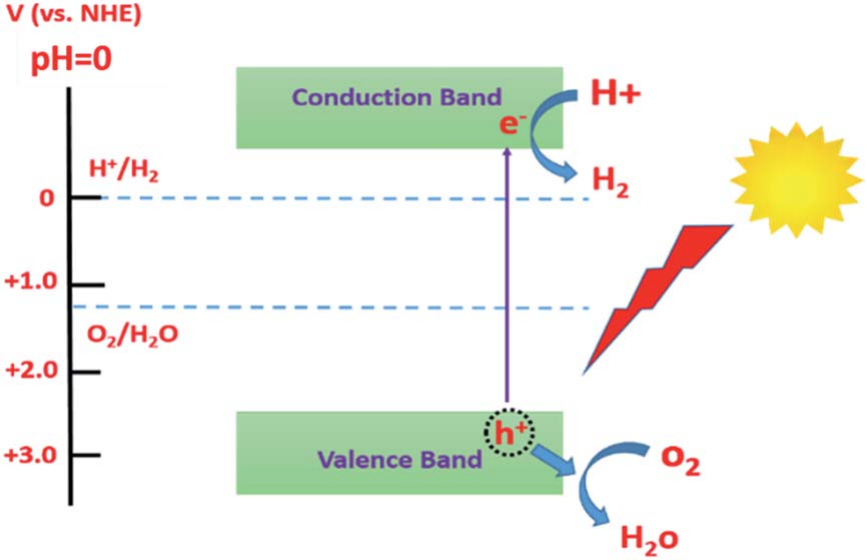
Semiconductor requirements for solar water splitting.

Environmental pollution and depletion of fossil fuels have become serious issues. In this regard, numerous studies have been carried out to utilize a renewable energy source that should be as efficient as fossil fuels but pollutant free. Harvesting solar energy has been utilized in photocatalytic hydrogen production *via* water splitting.^86^ Photocatalytic processes are reactions that can be accelerated or activated by means of the absorption of photons.^87^ Absorbed photons yield photogenerated electron/hole pairs that can derive certain redox reactions such as degradation of organic pollutants and water splitting. Among the photocatalytic materials, semiconductors are of great interest due to their potential applications in solar energy harvesting. However, for a semiconductor to allow a certain photocatalytic reaction, it must satisfy some criteria including a relatively small band gap to harvest as much energy as possible from the solar spectrum and convert these photons to well-separated charge carriers (e^−^/h^+^ pairs).^88,89^ It also needs to exhibit high chemical stability in aqueous electrolytes as well as being earth abundant to be cost-effective. Fig. 9 illustrates the conditions that should be satisfied by a semiconducting material for use in solar water splitting.^88^

In this regard, TiO_2_ has been considered as an outstanding photocatalyst owing to its high chemical stability, availability, low cost, and environmentally friendly nature. Despite all these advantages, TiO_2_ suffers from its restricted absorbance in the UV region of the solar spectrum as well as the fast recombination rate of the photogenerated charge carriers.^90,91^ In order to overcome these drawbacks, the morphology of TiO_2_ was modified to obtain one dimensional TiO_2_ nanotubes that can offer an enhanced photocatalytic performance due to the enhanced separation of photogenerated charge carriers by decoupling the direction of light absorption and charge carrier collection.^92^ Moreover, band gap engineering *via* doping, decoration and/or alloying with different elements (metals or non-metals) was reported to extend its light harvesting into the visible region of the solar spectrum.^93,94^

David *et al.*^95^ reported the impact of loading TNTs, fabricated *via* rapid breakdown anodization, with Pt, Pd and Ni nanoparticles on the efficiency of hydrogen generation *via* solar water splitting. The as-prepared TNTs were annealed at 450 °C for 3 h then sensitized with the metal nanoparticles through the chemical reduction approach using NaBH_4_. The XRD patterns showed that all the samples exhibited a pure anatase phase without any induced crystal structure modification. The metal nanoparticles were loaded on TNTs with two different concentrations, 5 wt% (denoted as PtA, PdA and NiA) and 10 wt% (denoted as PtB, PdB and NiB). The samples loaded with Pt and Pd nanoparticles exhibited enhanced hydrogen generation due to the created Fermi level of the metal just beneath the conduction band of the TNTs, resulting in an increased life time for the photogenerated charge carriers to drive the corresponding water splitting process. At high concentration of metal NP loading, the samples showed a decreased photocatalytic performance due to the agglomeration of the metal NPs at the active sites of the TNTs, thus preventing the penetration of light to these sites. On the other hand, sensitizing the TNTs resulted in deterioration of the hydrogen generation rate of the Ni sensitized TNTs compared to the pristine one. This behaviour was attributed to the created impurity level, which was far below the CB of the TNTs, making it difficult for the photogenerated electrons in the CB to be transferred to the Fermi level of the Ni NPs.^95^

**Fig. 10:**
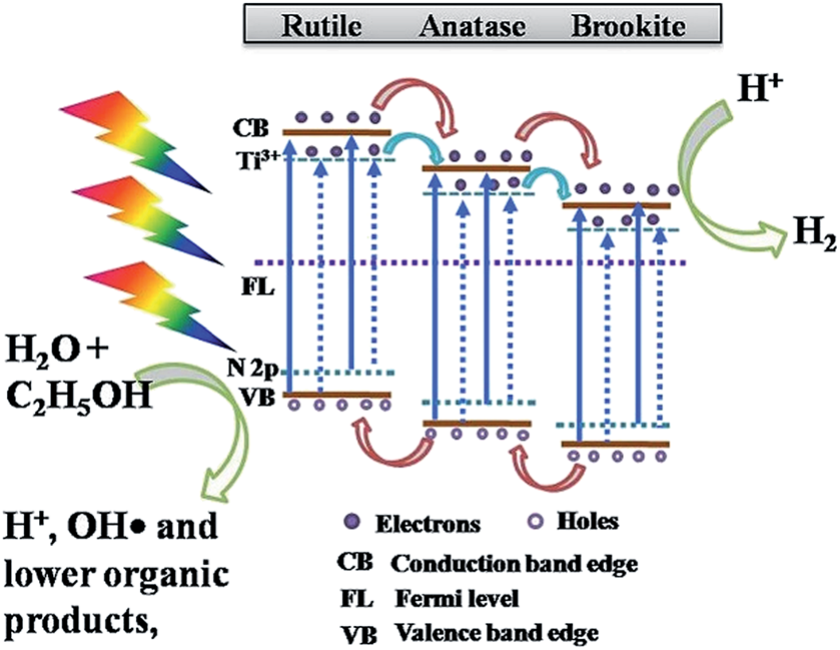
Schematic diagram of the charge transfer mechanism in 0.29 atomic% N-doped triphase TNTP. Reproduced from ref. 94 with permission from the Nature Publishing group under Creative Commons Attribution 4.0 International License.

For non-metal doping, Preethi *et al.* showed that N-doped triphase (anatase–rutile–brookite) TNTP exhibited a superior photocatalytic activity for solar water splitting compared to the pristine triphase TNTs. This enhancement was ascribed to engineering the band gap by N-doping from 3.06 eV down to 2.87 eV, which resulted in extending the photocatalytic activity into the visible region of the solar spectrum as illustrated by the charge transfer mechanism shown in Fig. 10. The pristine triphase TNTP was prepared *via* the rapid breakdown anodization method. While the N-doped sample was prepared by adding different concentrations of hydrazine hydrate to the electrolyte solution rather than annealing the pristine TiO_2_ in an NH_3_ atmosphere since annealing in an NH_3_ atmosphere resulted in the formation of N-doped biphase TNTs. The XRD patterns confirmed that both pristine and N-doped TNTP exhibited diffraction peaks that are indexed to the three phases (anatase–rutile–brookite). Also, it was illustrated that increasing the concentration of the N dopant induced phase transformation from brookite to anatase. The photocatalytic measurements revealed that doping TiO_2_ with a nitrogen concentration of 0.29 atomic percentage exhibited the best photocatalytic performance in hydrogen production (30.2 mmol g^−1^) *via* solar water splitting.^94^

## Energy storage applications

Due to the increased need for energy in our daily life, it is mandatory to fabricate long-lasting energy storage devices. In this regard, supercapacitors are considered the energy storage devices of the future. Electrochemical capacitors (EC) can store energy in the form of electrical charges.^96^ The materials used for supercapacitors can be divided into three main categories.^97–99^ First, materials that store energy in the form of electric double layers (EDLs), which are mainly carbon allotropes such as graphite, graphene, and carbon nanotubes. Second, materials that store energy through a fast-redox reaction from different chemical groups such as oxides, sulfides, nitrides, or conducting polymers.^100,101^ Third, composite materials of both active and double layer materials.^96,99^ In battery-like materials, the capacitance is subject to change over the potential window.^102^ The process of storing energy through a redox reaction is usually referred to as the “faradaic process”. In the faradaic process, a fast reversible redox reaction occurs at the surface of the electrode material resulting in adsorption of electrolyte ions and exchange of electron charges between the electrolyte and the electrode material.^96,103^ The surface of the material is the main factor that controls the adsorption of the ions and the charge exchange. Thus, the morphology of the material is a good subject to be studied as it affects the mechanism and the quality of the pseudocapacitor material.^103^

**Fig. 11:**
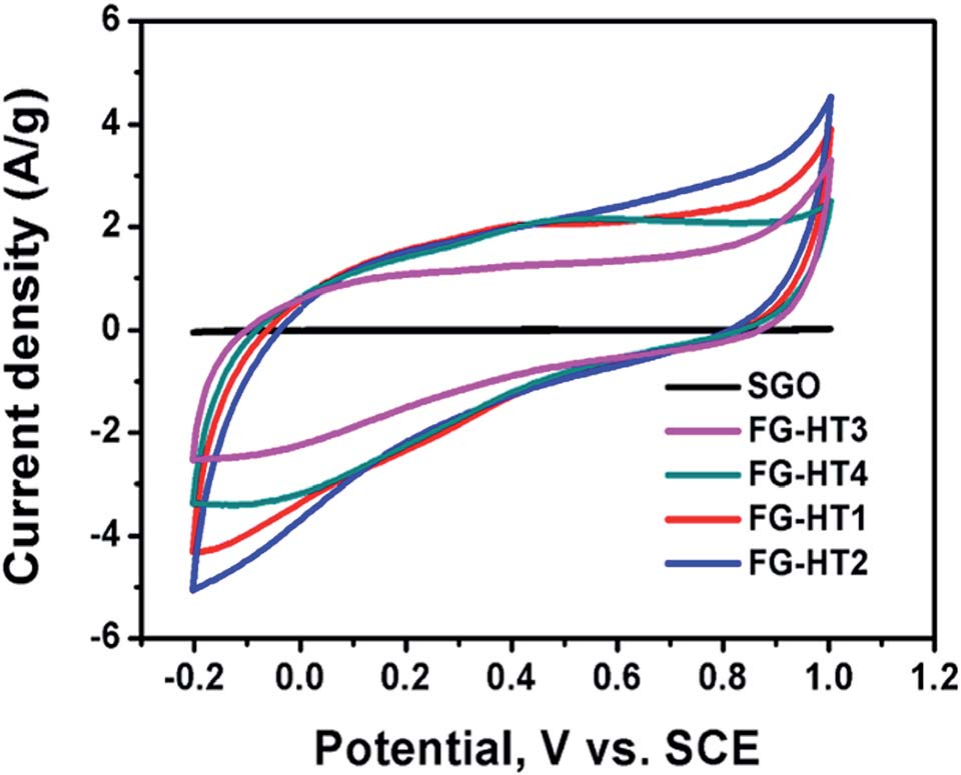
Cyclic voltammograms of spongy graphene oxide and hydrogenated TiO_2_ with different ratios of functionalized graphene oxide. Reproduced from ref. 117 with permission from the Royal Society of Chemistry.

Although TiO_2_ is a cheap and stable material that can undergo redox reactions, it has a relatively low conductivity, making it a poor target for supercapacitor applications. To this end several modifications have been adopted to benefit from the unique properties of TiO_2_ such as its large surface area.^83–87^ Moreover, composites of TiO_2_ nanotube arrays with carbon materials are getting great attention.^106–109^ TiO_2_ nanotube arrays usually exhibit a typical rectangular cyclic voltammogram (CV), indicating pseudocapacitive behaviour.^104,110^ In addition, it also exhibits minor EDL behaviour which is very beneficial for charge storage.^111^ Some studies suggest that intercalation of TiO_2_ with ions in the electrolyte might lead to battery-like behaviour.^112^ Meanwhile, TiO_2_ nanotube arrays have low capacitance due to their low conductivity, which motivates researchers to induce modifications to increase their capacitance.^105,110,112–114^ Among those modifications is the use of alternative methods to produce titania nanotubes in the powder form.^115^ To this end, Wu *et al.*^116^ used hydrogen plasma treatment for TiO_2_ nanotubes in order to enhance their capacitive properties. The prepared nanotubes were removed from the surface of Ti foil using adhesive tape then annealed in air at 450 °C. Sequentially, the obtained nanotubes were exposed to a plasma enhanced chemical vapour deposition chamber at 320 °C under vacuum. The hydrogen plasma was then introduced along with hydrogen gas flow. The resulting hydrogenated TiO_2_ showed a darker colour indicating more defects and it was suggested that the hydrogen atoms were used to passivate the dangling bonds in the shell layer. The phase of the resulting TiO_2_ was mostly anatase, which has higher electrical conductivity. The electrochemical properties of the hydrogenated titania were studied in a three-electrode system in 2 M Li_2_SO_4_ as the electrolyte, Pt foil as the counter electrode, and Ag/AgCl as the reference electrode. The resulting CV showed a quasi-rectangular shape with a potential window of −0.3 to 0.6 V, which indicates high EDL character. The CV curve of the plasma-treated titania was 7.2 times larger than that of the titania powder without treatment. The charge/discharge specific capacitance showed that plasma treatment greatly increased the capacitance of titania nanotubes. The increase in the capacitance was ascribed to the improvement of the conductivity of titania as a result of increasing the number of charge carriers due to the increasing Ti^3+^ sites. On the other hand, Dalia El-Gendy *et al.*^117^ have used a TiO_2_/spongy graphene composite for supercapacitor applications. The added graphene enhanced the capacitance of the hydrogenated TiO_2_ powder which reached 400 F g^−1^ at a 1 mV s^−1^ scan rate and increased the potential window in the positive potential region. The study showed that the TiO_2_ powder affected the behaviour of the cyclic voltammetry curves which deviated from the ideal rectangular shape of ideal EDL electrodes. On the other hand, the study showed that the more the functionalized graphene oxide added to the powdered TiO_2_, the higher the specific capacitance. Fig. 11 shows the enhancement of the spongy graphene capacitance with the addition of TiO_2_ and the enhancement of the TiO_2_ powder capacitance with increasing the ratio of the functionalized graphene.^117^ TiO_2_ powder also showed high performance upon its use in Li-ion batteries. It was shown that allowing TiO_2_ to self-crystallize and relax in its best structure gives the highest diffusion possibility of Li ions into the TiO_2_ crystals. The amorphous cubic structure of TiO_2_ showed a specific energy of 200 W h kg^−1^ at a specific power of 30 W kg^−1^ with high stability over 600 cycles.^118^

## Environmental applications

### Sensing applications

**Fig. 12:**
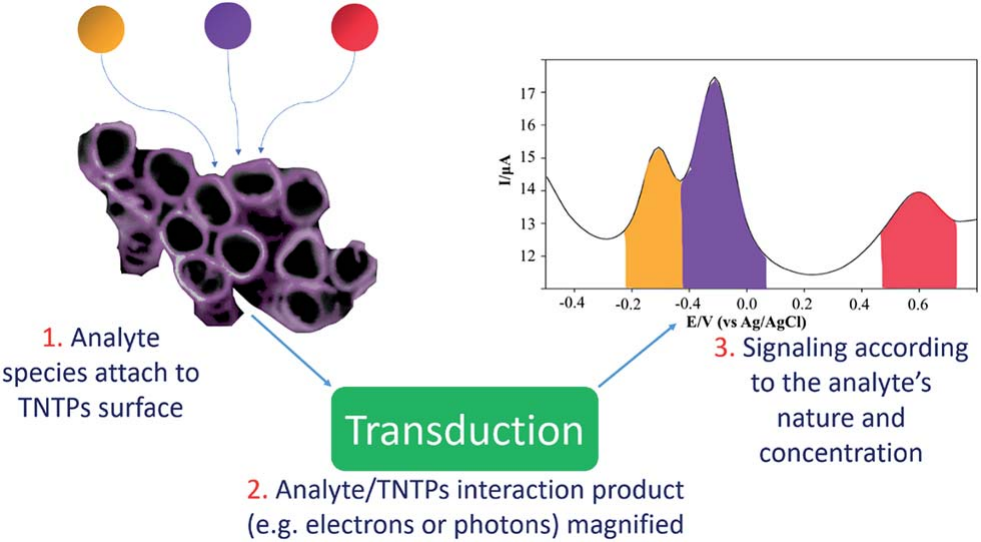
Schematic representation of the sensing mechanism on TNTP platforms.

Due to its chemical stability, biocompatibility, and remarkable catalytic properties,^119^ TiO_2_ nanotubes have been utilized in different applications, particularly in the powder form.^120^ One important example of these applications is sensing platforms, where TiO_2_ nanotubes can be used as a catalyst to bring all the target molecules of the analyte together on their surface to speed up the detection reaction, according to the sensing mechanism, see Fig. 12.^121^ In brief, the nanotubular structure of titania, acting as a supporting platform, helps to increase the specific surface area of the sensor and leads to a higher probability of interaction between the target molecules and the TNTP, especially if they are functionalized with another sensitive material.^122^ In this case, TNTP will also act as a scaffold to reduce the chances of agglomeration and increase the dispersity of the modifier, as it is usually added in minor amounts. It is worth mentioning that the thin wall thickness of TNTP plays an important role in the sensing mechanism by facilitating the pathways for charge collection after accumulation of the analyte species on the surface.^122^ Typically, some ions may attach to the nanotubular nozzles, while some others can be embedded onto the tubular surface. Some ions may even infiltrate inside the tubes to adsorb on the inner tubular walls.^122^ All this increases the possibility for the ionic species to be adsorbed and for their charges to be collected on the one-dimensional structure of TNTP. Accordingly, a TNTP-based sensing platform can exhibit high specificity and selectivity toward the species of interest, especially with the enhanced charge collection that the tubular geometry can induce.^29^ The sensing strategy itself can be utilized for different purposes. For instance, Abdullah *et al.* have used TiO_2_ nanotube powder in a composite with reduced graphene oxide (RGO) for an environmental approach. The sensing platform was designed against Hg(ii), Cu(ii), and Mn(ii) ions as toxic pollutants in the aquatic environment. The study achieved a limit of detection (LOD) in the ppt level and showed how TiO_2_ nanotubes enhanced the electrocatalytic activity of the composite *via* acting as a template to minimize the agglomeration of RGO, making use of its low band gap character.^122^ TiO_2_ nanotubes have also been employed to enhance the detection of other metals such as Fe(iii) and La(iii) by promoting the sensitivity and the ion uptake of the sensors' adsorption sites.^123,124^ Additionally, pharmaceutical analyses have utilized TiO_2_ nanotubes in composites to electrochemically determine the concentration of certain drugs such as metformin and benzocaine.^125^ The LODs of both studies were as low as 3 nM each, which indicates the distinctive electrochemical properties of TiO_2_ nanotubes for such applications. Furthermore, TiO_2_ nanotube powder has been widely used for gas sensing applications. This includes a variety of gases such as hydrogen, acetone, and hydrogen peroxide, either using pristine or metal loaded TiO_2_ nanotube powder.^126^ Recently, a study by David *et al.* has proved the enhanced H_2_O_2_ sensing properties of TiO_2_ nanotube powder especially when loaded with Pt. This granted the feasible pathways for electron transfer and enhanced the irreversible nature of the electrochemical reaction.^126^ This would pave the way for further functionalization of TiO_2_ nanotube powder using more cost-effective and earth-abundant metals in the near future.

### TNTP for pollutant degradation applications

Due to their unique properties, TNTs have been used extensively in solid phase extraction and degradation of various pollutants in environmental and industrial applications. To be more specific, residual dyes resulting from several industries are perceived as highly undesirable organic pollutants that result in huge quantities of wastewater.^127^ For the time being, it is very crucial to turn such wastewater into more useable resources for drinking or irrigation after either degradation or removal of pollutants. For example, Table 2 shows the contribution of reactive dyes to wastewater production due to their low fixation rates in the textile industry.^128^

**Table 2:** Mass of dye wastewater from different types of textile dyes

Types of textile dyes	Acid	Reactive	Disperse	Direct	Vat	Basic	Sulfur
Mass of dye water (1000 tons)	20	58	18	20	8	3	40

**Fig. 13:**
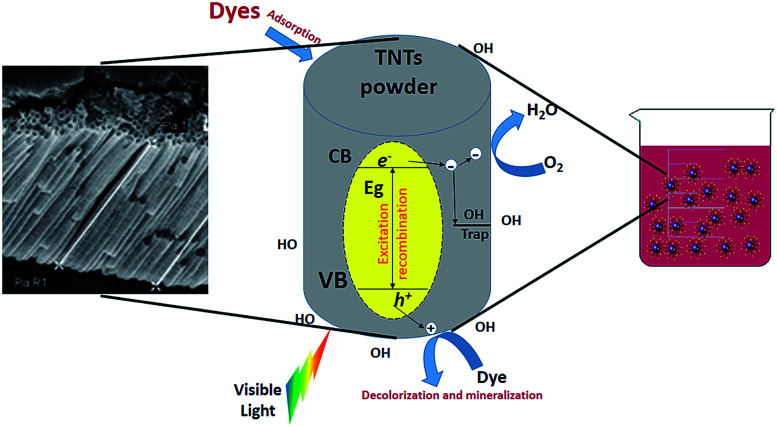
The principal mechanism for using TNTP in the process of environmental disinfection.

Nonbiodegradable organic dyes may cause wastewater to have high toxicity to humans, aquatic life and the environment. Their high colour intensity may block sunlight from passing through water which creates restriction for aquatic diversity. It is widely acknowledged that the some of the released aromatic compounds in wastewater are considered toxic, carcinogenic, or mutagenic.^129–131^ Hence, the use of such contaminated wastewater may cause different dermal and respiratory diseases in humans.^132^ The obstreperous nature of dye wastewater treatment arises from the fact that organic compounds cannot be digested aerobically nor naturally degraded by light.^133^ However, photocatalytic degradation of organic dyes by TNTs has been of interest due to their high photon absorption through a large number of active sites.^134–136^ In addition, the unique one-dimensional aligned structure helps in increasing photocatalytic efficiency through vertical charge transport resulting in little loss at grain boundaries through recombination.^137–139^ The degradation mechanism depends on the electron and hole production upon TNT exposure to light. The produced electrons and holes help reduce O_2_ and oxidize H_2_O molecules, respectively. The formed species, typically oxide ions and hydroxyl radicals, have a powerful effect toward organic pollutants, degrading them into their primary molecules such as CO_2_.^66^ A similar mechanism is proposed for the antibacterial effect of TNTP which will be discussed later on. Fig. 13 describes the principal mechanism for using TNTP in the process of environmental disinfection. In 2005, Quan *et al.* explained the higher photo-electrochemical degradation of pentachlorophenol by TNTs in comparison with ordinary TiO_2_ nanoparticles due to their larger kinetics constant.^139^ In addition, TNTs have also been proved to exhibit twice the degradation efficiency of TiO_2_ nanoparticles for acid orange dye.^140,141^ In order to reduce some of the common limitations of TNTs such as a wide band gap and ability to function effectively only in the UV region, significant efforts have been made to enhance TNTs’ photocatalytic activity by anionic/cationic doping or other techniques.^142,143^ Different researchers proposed binary systems since they can diminish recombination while accumulating both holes and electrons in two dissimilar layers to enable charge carrier separation.^144–147^

Although introducing impurity levels by cationic dopants might restrict migration of charge carriers if the optimum value is exceeded,^126^ it can enhance response in the visible light spectrum. This can be ascribed to the decrease in the lifetime of the electron–hole pairs which was explained through doped sites that may act as recombination sites for charge carriers. Similarly, anion-doped TiO_2_ shows a smaller bandgap than ordinary TiO_2_ which is attributed to the higher potential energy of nanometals that form a new VB closer to the CB. It is believed that anion doping can enhance the photocatalytic activity of TNTs than cationic doping in the visible region, due to the impurity states close to the VB reducing recombination.^127^

Compared to the TNT arrays that are directly attached to the metal substrate, TNTP can exhibit a superior photocatalytic performance in the degradation of organic pollutants. This enhancement was ascribed to the higher surface area of the latter. As annealing at high temperatures of the TNT arrays attached to the Ti metal substrate leads to crystallite growth in the TNT walls resulting in increased tube wall thickness and subsequently decreased surface area.^14^ Another drawback that can lower the photocatalytic activity of TNT arrays attached to the Ti substrate is that the transformation from the anatase phase to the rutile one upon annealing above 550 °C.^147–149^ Destabilizing the anatase phase of the TNTs results in shrinking of their photocatalytic activity.^150,151^

**Fig. 14:**
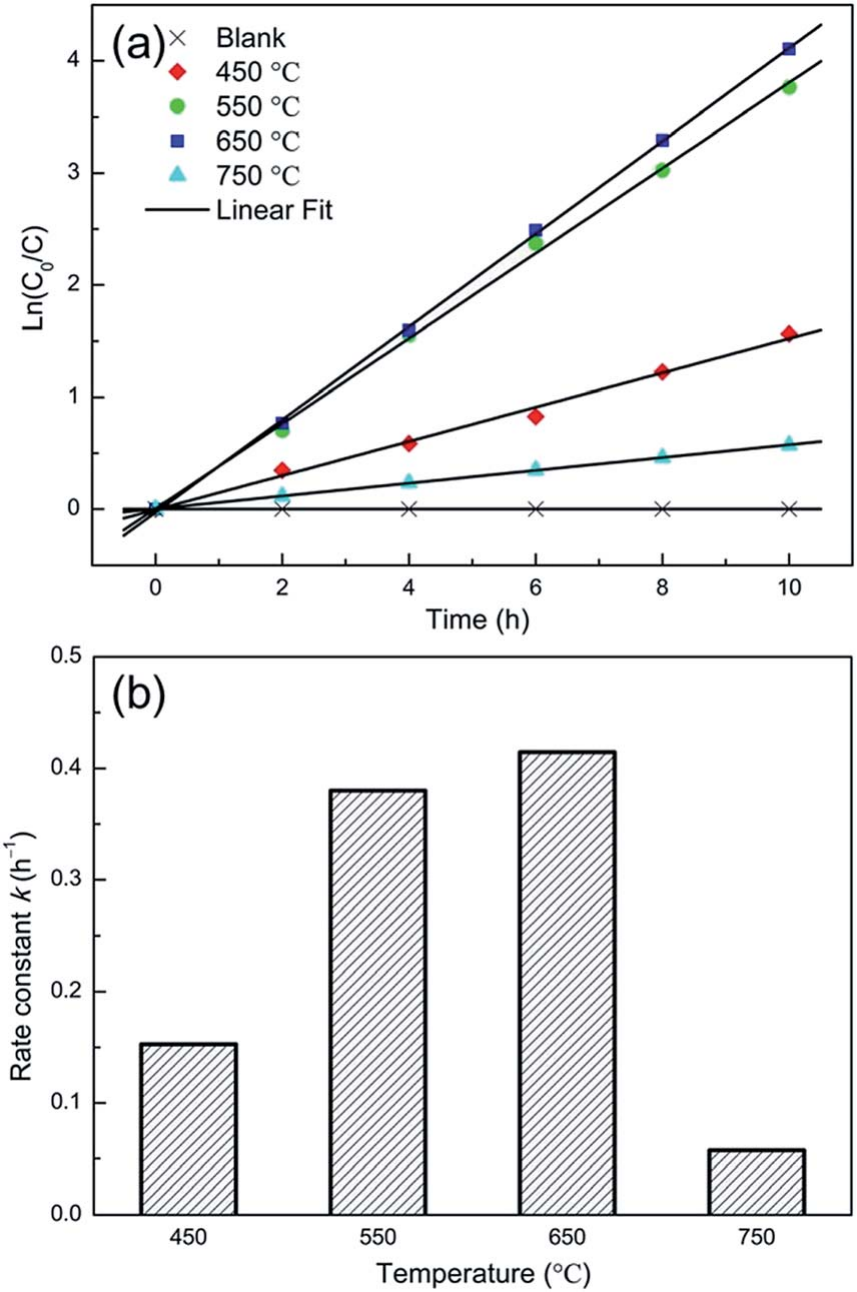
(a) Time dependent MB concentration showing the photocatalytic decomposition kinetic behaviour of the NT powders obtained at various annealing temperatures; (b) the photocatalytic rate constant. Reproduced from ref. 14 with permission from the Springer Publishing group under Creative Commons Attribution 4.0 International License.

Jia *et al.*^14^ studied the effect of annealing temperature on the crystal structure and the photocatalytic performance of the TNTP. The TNTs were prepared *via* the anodization technique followed by sonication in ethanol in order to remove the nanotube layer and then the as-prepared TNTP were subjected to different annealing temperatures (450, 550, 650 and 750 °C) for 2 h in air. The SEM and XRD patterns showed that all the samples conserved the tubular morphology and the anatase crystal structure upon annealing up to 750 °C, respectively. Despite the decreased specific surface area of the TNTs upon increasing the annealing temperature up to 750 °C, the samples showed enhanced photocatalytic degradation of methylene blue upon increasing the annealing temperature up to 650 °C. This indicates the superior effect of the enhanced crystallinity compared to the effect of the specific surface area as shown in Fig. 14.^14^

Liang *et al.*^93^ demonstrated the effect of doping TNTP with cobalt ions on the photocatalytic degradation of methylene blue under UV irradiation. The co-precipitation method followed by hydrothermal treatment was used to fabricate un-doped and Co-doped TNTP. The as-prepared samples were annealed at 450 °C for 3 h. The XRD patterns were indexed to the anatase phase and confirmed that doping with cobalt ions at low concentrations does not affect the crystal structure of the TNTs. Doping TNTs with Co ions at concentrations up to 1.3% increased the photocatalytic degradation rate of methylene blue up to 97.2% compared to the un-doped TNTs’ 80.6% under UV irradiation.^93^

## Conclusions & future perspectives

TNTP are a type of semiconducting material that can offer advantages such as feasible synthesis, low cost, and promising performance for a variety of applications. This review recapitulates the cutting-edge knowledge about TNTP developed and experimentally tested. Fabrication methods such as ultra-sonication, hydrothermal processing, and rapid breakdown anodization have been summarized and the properties of the produced TNTP were further discussed. Effects of synthesis techniques and defect structures on TNTP for biological applications were reviewed. More investigation is needed to evaluate how TNTP can be better utilized as drug carriers and sensing substrates where TiO_2_ is currently a predominant platform. Based on the biological advantages of TNTP, using them for antibacterial approaches has been discussed. The review also demonstrated the auspicious performance of TNTP for energy conversion applications. This is expected to be more effective upon better fundamental understanding and control of TNTP structural parameters such as anchoring, sensitization, decoration and functionalization. The effect of TNTP preparation conditions on their capacitance and organic degradation applications has also been overviewed. Comparative studies between TNTP and TNT arrays would be useful to assess the former's efficiency when produced with other fabrication techniques and under other treatment conditions such as annealing parameters, defect formation, and phase change. One of the important future insights is to synthesize mixed oxide nanotube powder to enhance the optical, electrical, and electrochemical performance of TiO_2_ nanotubes. Another future trend could be the development of various protocols to dope the TiO_2_ nanotube powder with foreign elements for various applications.

## Conflicts of interest

The authors declare no conflict of interest.

## Supplementary Material
